# The Protective Role of Fucosylated Chondroitin Sulfate, a Distinct Glycosaminoglycan, in a Murine Model of Streptozotocin-Induced Diabetic Nephropathy

**DOI:** 10.1371/journal.pone.0106929

**Published:** 2014-09-05

**Authors:** Conrado L. R. Gomes, Cristina L. Leão, Carolina Venturotti, André L. Barreira, Gabriela Guimarães, Roberto J. C. Fonseca, Rodrigo S. Fortunato, Paulo A. S. Mourão, Alvimar G. Delgado, Christina M. Takiya, Maurilo Leite

**Affiliations:** 1 Serviço e Disciplina de Nefrologia, Departamento de Clínica Médica and Hospital Universitário Clementino Fraga Filho, Universidade Federal do Rio de Janeiro, Rio de Janeiro, Brazil; 2 Instituto de Ciências Biomédicas, Universidade Federal do Rio de Janeiro, Rio de Janeiro, Brazil; 3 Laboratório de Tecido Conjuntivo, Instituto de Bioquímica Médica and Hospital Universitário Clementino Fraga Filho, Universidade Federal do Rio de Janeiro, Rio de Janeiro, Brazil; 4 Laboratório de Radiobiologia Molecular, Instituto de Biofísica Carlos Chagas Filho, Universidade Federal do Rio de Janeiro, Rio de Janeiro, Brazil; INSERM, France

## Abstract

**Background:**

Heparanase-1 activation, albuminuria, and a decrease in glomerular heparan sulfate (HS) have been described in diabetic nephropathy (DN). Glycosaminoglycan (GAG)-based drugs have been shown to have renoprotective effects in this setting, although recent trials have questioned their clinical effectiveness. Here, we describe the effects of fucosylated chondroitin sulfate (FCS), a novel GAG extracted from a marine echinoderm, in experimentally induced DN compared to a widely used GAG, enoxaparin (ENX).

**Methods:**

Diabetes mellitus (DM) was induced by streptozotocin in male Wistar rats divided into three groups: DM (without treatment), FCS (8 mg/kg), and ENX (4 mg/kg), administered subcutaneously. After 12 weeks, we measured blood glucose, blood pressure, albuminuria, and renal function. The kidneys were evaluated for mesangial expansion and collagen content. Immunohistochemical quantifications of macrophages, TGF-β, nestin and immunofluorescence analysis of heparanase-1 and glomerular basement membrane (GBM) HS content was also performed. Gene expression of proteoglycan core proteins and enzymes involved in GAG assembly/degradation were analyzed by TaqMan real-time PCR.

**Results:**

Treatment with GAGs prevented albuminuria and did not affect the glucose level or other functional aspects. The DM group exhibited increased mesangial matrix deposition and tubulointerstitial expansion, and prevention was observed in both GAG groups. TGF-β expression and macrophage infiltration were prevented by the GAG treatments, and podocyte damage was halted. The diabetic milieu resulted in the down-regulation of agrin, perlecan and collagen XVIII mRNAs, along with the expression of enzymes involved in GAG biosynthesis. Treatment with FCS and ENX positively modulated such changes. Heparanase-1 expression was significantly reduced after GAG treatment without affecting the GBM HS content, which was uniformly reduced in all of the diabetic animals.

**Conclusions:**

Our results demonstrate that the administration of FCS prevented several pathological features of ND in rats. This finding should stimulate further research on GAG treatment for this complication of diabetes.

## Introduction

Diabetic nephropathy (DN), the leading cause of end-stage chronic kidney disease worldwide [1], has a complex pathogenesis that is not completely understood [2]. The glomerular filtration barrier (GFB) is composed of fenestrated endothelium, a glomerular basement membrane (GBM), and podocytes. Disturbances in the GFB result in the loss of its remarkable permselectivity [3] and increasing levels of albuminuria, a sign of diffuse endothelial damage [4] and a surrogate marker of early DN [5]. Early findings have indicated that the GBM, with its highly anionic heparan sulfate (HS) proteoglycan (PG) content, is an important charge barrier to albumin filtration [6]–[8]. However, recent evidence against this hypothesis has been presented [9]–[13]. For instance, GBM HS degradation via the infusion of the bacteria-degrading enzyme heparinase III in rats resulted in the loss of anionic sites in the GBM but did not result in proteinuria for up to 48 h [9].

Despite these ongoing controversies, the increased expression and activity of heparanase-1, an endo-β(1→4)-D-glucuronidase that cleaves HS chains, has been demonstrated in DN and other proteinuric renal diseases [14]–[20]. Increased heparanase-1 expression has been described in a biopsy study of DN patients compared to patients with other glomerular diseases [15]. In addition, the urinary excretion of heparanase-1 has been shown to be increased in type I and II diabetic patients with albuminuria [16], [17]. Furthermore, heparanase-1 gene knockout mice have been shown to be significantly protected against the pathological renal consequences of DM compared to wild-type mice [18].

More than two decades of intense research has shown that glycosaminoglycan (GAG)-based compounds have therapeutic potential in several renal diseases [21]–[23], especially DN [24]–[28]. The possible mechanisms for heparin and GAG-based drugs in this setting include their ability to block heparanase-1 synthesis and activity [26], [27] and to inhibit TGF-β at the transcriptional level [25], [28], [29]. Furthermore, GAG-based compounds have been shown to reduce macrophage infiltration and proteinuria in puromycin nephrosis [23]. The initial clinical experience of treating DN with sulodexide (a combination of fast-moving heparin and dermatan sulfate) has been promising [30]–[32]. However, the recent negative outcomes of two large clinical trials [33], [34] have created some skepticism regarding its effectiveness. Thus, given concerns regarding the trial designs, and also the sulodexide formulations used in those studies [35] along with the experimental and clinical evidence favoring GAG use, we were motivated to investigate the effects of a distinct GAG formulation, fucosylated chondroitin sulfate (FCS), in a murine model of DN. FCS is extracted from the marine invertebrate, *Ludwigothurea grisea* and is composed of a central carbohydrate core similar to mammalian chondroitin sulfate. However, it contains unique sulfated α-L-fucopyranose branches linked to position 3 of the D-glucuronic acid residue. These branches provide this compound with distinct biological properties that are responsible for its anticoagulant, anti-metastatic, and anti-inflammatory activities, as demonstrated previously by our group [36]–[38].

The present study was designed to evaluate the effects of FCS in a model of streptozotocin (STZ)-induced type I DM in rats, and these effects were compared to the well-established, widely available, and commonly prescribed GAG, enoxaparin (ENX).

## Subjects and Methods

### Animals

Twenty 10-week-old male Wistar rats weighing 210–270 g were housed at a controlled temperature (23°C±2°C) and relative humidity (50%–60%) under a 12-h light/dark cycle. The animals were fed water and standard rat chow ad libitum. All of the experimental procedures were approved by the Committee for Experimental and Animal Ethics at the Federal University of Rio de Janeiro (Permit Number: HUCFF011) and were conducted in accordance with “The Guide for Care and Use of Laboratory Animals” (available online at http://www.nih.gov).

### Experimental protocol

Diabetes was induced in 15 rats using a single tail vein injection of 65 mg/kg STZ (Sigma Chemical, St. Louis, MO, USA) in 0.05 M citrate buffer (pH 4.5). A control group of 5 rats received citrate buffer only. Diabetes was confirmed 48 h later by measuring blood glucose levels by tail vein puncture followed by reflectometric analysis (Accu-Chek Active System, Roche, Basel, Switzerland). Values greater than 300 mg/dL were considered positive for diabetes. To prevent excessive weight loss and ketoacidosis, daily injections of NPH insulin (1–4 U) were administered. Eight weeks after DM induction, the rats were randomly divided into 3 groups consisting of 5 rats each: DM, untreated diabetic rats; FCS, diabetic rats receiving 8 mg/kg fucosylated chondroitin sulfate subcutaneously (SC); and ENX, diabetic rats receiving 4 mg/kg enoxaparin SC (Heptron Cellopharm/Aspen Pharmacare, Durban, South Africa). A fourth group of 5 rats without DM was used as a control. The GAGs were administered daily for 6 weeks and then every other day for an additional 6 weeks. The extraction, purification, and safety profile of FCS has been previously described [36].

Blood glucose was monitored weekly and was maintained at approximately 400–500 mg/dL. At baseline and before euthanasia, all of the animals were placed in metabolic cages to collect 24-hour urine samples. Urinary albumin excretion was determined by ELISA (NephRat Kit, Exocell, Philadelphia, PA, USA). Creatinine and blood urea nitrogen levels were determined using enzymatic methods. The estimated glomerular filtration rate (eGFR) was determined from the creatinine clearance and calculated as urinary creatinine (mg/dL) × urine volume (mL)/serum creatinine (mg/dL) ×1,440 (min). The results were then corrected for the body weight and expressed in mL/min/100 g. The systolic blood pressure (SBP) was measured using the tail cuff method.

### Tissue preparation

After 20 weeks of induced DM, the rats were euthanatized under deep anesthesia through abdominal cavity dissection and perfusion with 0.9% sterile saline and heparin (5 U/mL) via left ventricular puncture. After perfusion, the right kidney was excised, weighed, and stored. Subsequent cardiac perfusion with 4% paraformaldehyde was performed to fix the remnant left kidney for histological analysis. After fixation, the left kidney was excised, weighed, and post-fixed at room temperature in 10% formaldehyde for 24 h.

### Histopathological and immunohistochemical studies

After fixation, the left kidney was embedded in paraffin. The kidney samples were sectioned at 3-µm intervals and stained with periodic acid Schiff reagent (PAS). For the total interstitial collagen estimation, Sirius Red staining was performed. Masson’s trichrome staining was used for the descriptive morphological analyses.

Immunohistochemical analysis was performed on 3-mm-thick, paraffin-embedded kidney sections. After dewaxing and rehydrating, the endogenous peroxidase was quenched for 15 min with H_2_O_2_ in methanol. Heat-mediated antigen retrieval and enzymatic techniques were preformed according to the specific antibody. A blocking step was performed using 5% bovine serum albumin (BSA) in PBS and normal rat serum, followed by a blockage of the endogenous biotin (Dako Biotin Blocking System, Glostrup, Denmark). Primary antibodies against specific antigens were then incubated overnight at 4°C in a humidified chamber. The sections were washed in 0.25% PBS-Tween solution for 5 min. After incubation with secondary antibodies, the samples were visualized using the Dako LSAB2 HRP-system kit (Dako, Glostrup, Denmark) with diaminobenzidine (Liquid DAB, Dako, Glostrup, Denmark) as a chromogenic substrate. Macrophages were detected using a monoclonal mouse ED-1 anti-rat antibody (1∶100; AbD Serotec, Raleigh, NC, USA). For the TGF-β analysis, a pan-specific anti-TGFβ antibody was used (1∶30; R&D System, Minneapolis, MN, USA). For estimating podocyte damage, a monoclonal antibody against nestin (1∶100; AbD Serotec, Raleigh, NC, USA), a constitutive protein in mature podocyte cytoskeletons [39], was used.

### Heparanase-1 and glomerular basement membrane heparan sulfate immunofluorescence analyses

To investigate the GBM HS content and the expression of heparanase-1, the following antibodies were used: JM403, an anti-HS antibody that recognizes HS domains containing N-unsubstituted glucosamine residues in the GBM (1∶30; AMS Biotechnology, Abingdon, UK), and HPA-1 M-45 anti-heparanse-1 antibody (1∶50; Santa Cruz Biotechnology, Santa Cruz, CA, USA). After dewaxing and rehydrating, the sections were preincubated with 5% BSA to prevent nonspecific binding. After endogenous peroxidase inhibition with H_2_O_2_ in methanol, antigen retrieval was performed by microwave followed by enzymatic methods (0.1% trypsin solution for HPA-1 and 0.1% trypsin followed by chondroitinase ABC [0.25 U/mL] for JM403). Nonspecific binding was blocked by incubation with 60% normal goat serum in 50 mM Tris 150 mM NaCl buffer (TBS) (pH 7.6). Endogenous biotin was blocked as previously described, and the primary antibodies were then incubated overnight. After washing with 0.05% TBS-Tween, the secondary antibody was added, and a Dako LSAB2 HRP-system kit (Dako, Glostrup, Denmark) was used. Then, the histological sections were incubated with a tyramide signal amplification kit (TSA Plus Fluorescence, PerkinElmer, Shelton, CT, USA) conjugated with a fluorophore (tetramethylrhodamine) to amplify antigen detection. The slides were mounted in VectaShield (Vector Laboratories, Burlingame, CA, USA) and visualized using an Eclipse E800 epifluorescence microscope (Nikon, Tokyo, Japan).

### Histomorphometry

For histomorphometry, an image analysis system consisting of a light microscope (Eclipse E800, Nikon, Tokyo, Japan) and a digital camera (Evolution, Media Cybernetics Inc., USA) using the Q-Capture 2.95.0 graphic interface software (version 2.0.5; Quantitative Imaging, Surrey, BC, Canada) was used. High-quality images (2048×1536 pixels buffer) were obtained and analyzed using the Image Pro Plus software (version 4.5.1; Media Cybernetics, Rockville, MD, USA).

### Mesangial expansion, tubulointerstitial area, and collagen deposition

From each animal in all groups, 100 glomeruli were stained by the PAS technique and images were captured using a 20× objective lens. The PAS-positive percentage of the glomerular tuft area corresponding to the mesangial axis (excluding the nuclei) was then determined [40]. The renal interstitium was defined as the space not occupied by glomeruli, tubules, or vessels [22]. Using a 40× objective lens, 20 consecutive fields were photographed, and the results were expressed as the ratio of the interstitial area to the total area. In addition, 20 fields captured by a 40× objective lens were analyzed for interstitial fibrosis and quantified as the area occupied by intertubular collagen fibers stained with Sirius Red. All of the evaluations were performed by a single observer.

### Macrophage (ED-1), TGF-β, and nestin surface density quantifications

The areas stained with the ED-1, TGF-β, and nestin antibodies were used to obtain 20 consecutive photomicrographs of the glomeruli (20× objective lens) or interstitial areas (40× objective lens). Semi-quantification analyses were performed as described. The results represent the surface densities for the entire field.

### Heparanase-1 and heparan sulfate quantification

Thirty glomeruli from each animal were captured using a 20× objective lens and were analyzed independently by 2 observers, who showed a high result correlation (r = 0.5684, *p<*0.0001, Pearson’s correlation). The results are expressed as a semi-quantitative score as follows: for heparan sulfate, the loss of GBM linearity staining was scored on a scale of 0 to 5, where 5 represents ≥76% loss, 4 = 51%–75% loss, 3 = 26%–50% loss, 2 = 11%–25% loss, 1 = <10% loss, and 0 =  no loss. For HPA-1, the glomerular staining intensity was also scored on a scale of 0 to 5, where 0 =  no staining, 1 = <10%, 2 = 11%–25%, 3 = 26%–50%, 4 = 51%–75%, and 5≥76% positivity.

### Real-Time Polymerase Chain Reactions

RNA was isolated from snap frozen renal cortical tissue from control and treated animals using Tryzol Reagent (Qiagen, Valencia, CA, USA). Following DNAse treatments and reverse transcription reactions, TaqMan quantitative real-time polymerase chain reactions were performed according to the manufacturer’s instructions, using primer sets for the proteoglycan core proteins and enzymes involved in GAG assembly and degradation (Accession numbers for TaqMan primer/probe sets: Gusb: Rn00566655_ml; Decorin: Rn01503161_m1; Perlecan: Rn01515780_g1; Versican: Rn01493755_m1; Glypican-1: Rn00578072_m1; Syndecan-4: Rn00561900_m1; Chondroitin sulfate synthase-1: Rn01478125_m1; N-deacetylase/N-sulfotransferase: Rn01491301_m1; Agrin: Rn00598349_m1; Collagen XVIII: Rn01428995_m1; Heparanase-1: Rn01428995_m1; heparan sulfate-3-O-sulfotransferase 1: Rn00584544_s1; available at: https://www.lifetechnologies.com/us/en/home/life-science/pcr/real-time-pcr/real-time-pcr-assays/taqman-gene-expression.html; Life Technologies, Carlsbad, CA, USA). GUSb was used as an internal control.

### Statistical analysis

For the statistical analysis, we used the GraphPad Prism software (GraphPad Software, La Jolla, CA, USA), version 5.01. Comparisons between groups were performed using one-way analysis of variance with Tukey’s post-test, as appropriate. The results are expressed as the means±SEMs. The values were considered to be statistically significant when *p<*0.05.

## Results

### Functional aspects

**Figure 1:**
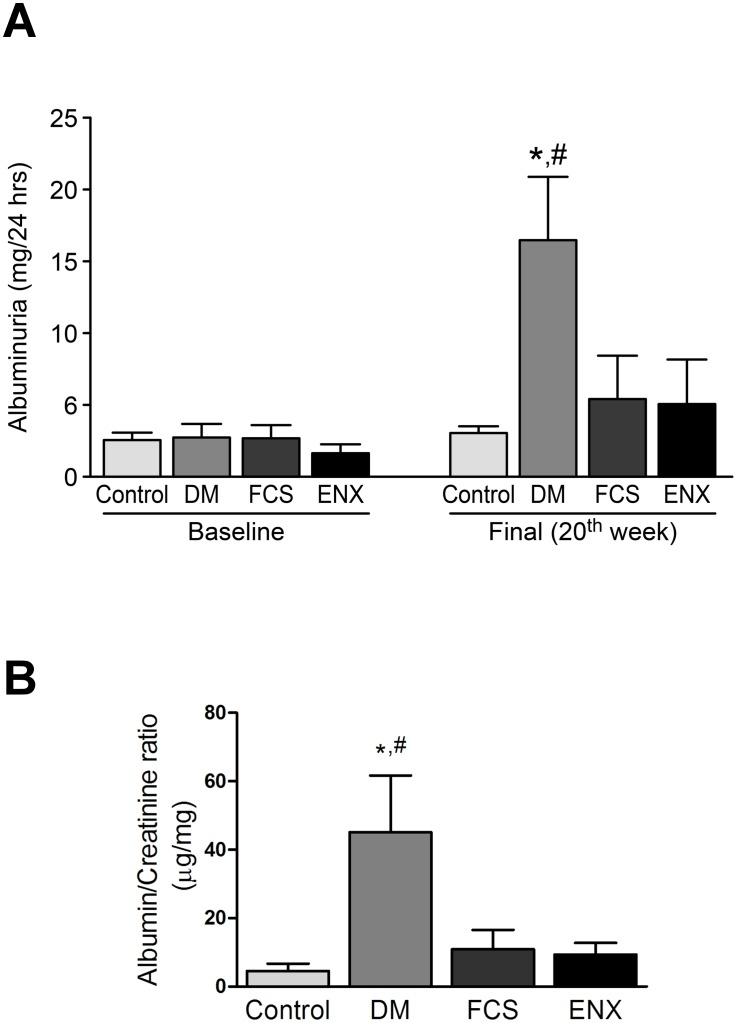
The time course for the urinary albumin excretion rate after 20 weeks of the study. A significantly increased level of albuminuria was evident in the DM group compared to the control group, with prevention in the DM rats treated with FCS and ENX. In A, Total albumin excretion in 24 hours (miligrams), at baseline and at the end of the twentieth week and in B, albumin (micrograms) to creatinine (miligrams) ratio in a random urinary sample at the end of the experiment. The data represent the means ± SEMs obtained from 5 rats per group. *p<0.01 DM vs. the baseline groups and final control group, #*p<*0.05 DM vs. the final GAG groups.

There were no differences in glycemic control among the DM groups during the study period (Table 1). All of the diabetic animals gained weight over time, although to a lesser extent than the nonDM control animals. At the end of the experiment, we observed higher kidney/body weight ratios and urinary volumes in all of the DM groups compared to the control animals. However, no differences were observed between the DM groups. Renal function remained stable in all groups. There was a trend toward higher blood pressure and eGFR in the DM group compared to the DM rats that received one of the two preparations of GAGs, but this difference was not statistically significant. The DM animals had significantly more advanced albuminuria than the controls, and significantly reduced albuminuria was observed in both the FCS and ENX groups (*p<*0.05) (Fig. 1; Table 1).

**Table 1:** Functional parameters at the end of the experiment.

Parameter	Control	DM	FCS	ENX	*p* value
Blood glucose(mg/dL)	97±1.2	498±10.4	530±10.7	531±13.1	<0.0001*
BWt (g)	492±47.6	335.8±34.1	345±46.7	326±40.6	<0.01*
RWt/100 g BWt	0.62±0.05	1.19±0.07	1.2±0.07	1.3±0.09	<0.0001*
SBP (mm Hg)	108±5.3	129±6	117.±7	118.±9.4	0.247
Urinary Volume(mL/24 hrs)	10,8±3	55±10,9	32±10,4	34±11,2	<0.01*
SCr (mg/dL)	0.7±0.14	0.7±0.14	0.78±0.15	0.68±0.15	0.92
eGFR(mL/min/100 g BWt)	1.6±0.37	3.3±1.03	2.2±0.27	2.6±0.6	0.35
Albuminuria(mg/24 hrs)					
Baseline	2.2±0.5	2.7±0.9	2.7±0.9	1.6±0.7	
Final	3.04±0.4	16.4±4.3**	5.3±3.05	5.06±3.1	<0.05#
Albumin/Creatinineratio (µg/mg)	4,58±2,1	45,09±16,6**	10,87±5,6	9,38±3,4	<0,05#

The results are presented as the means ± SEMs. BWt, body weight; RWt, renal weight; SBP, systolic blood pressure; SCr, serum creatinine; eGFR, estimated glomerular filtration rate; DM, diabetic group; FCS, diabetic group treated with fucosylated chondroitin sulfate; ENX, diabetic group treated with enoxaparin;

*all of the diabetic groups vs. control;

# DM vs. FCS and ENX groups;

**p<0.01 DM vs. control.

### Mesangial expansion, tubulointerstitial area and collagen deposition

**Figure 2:**
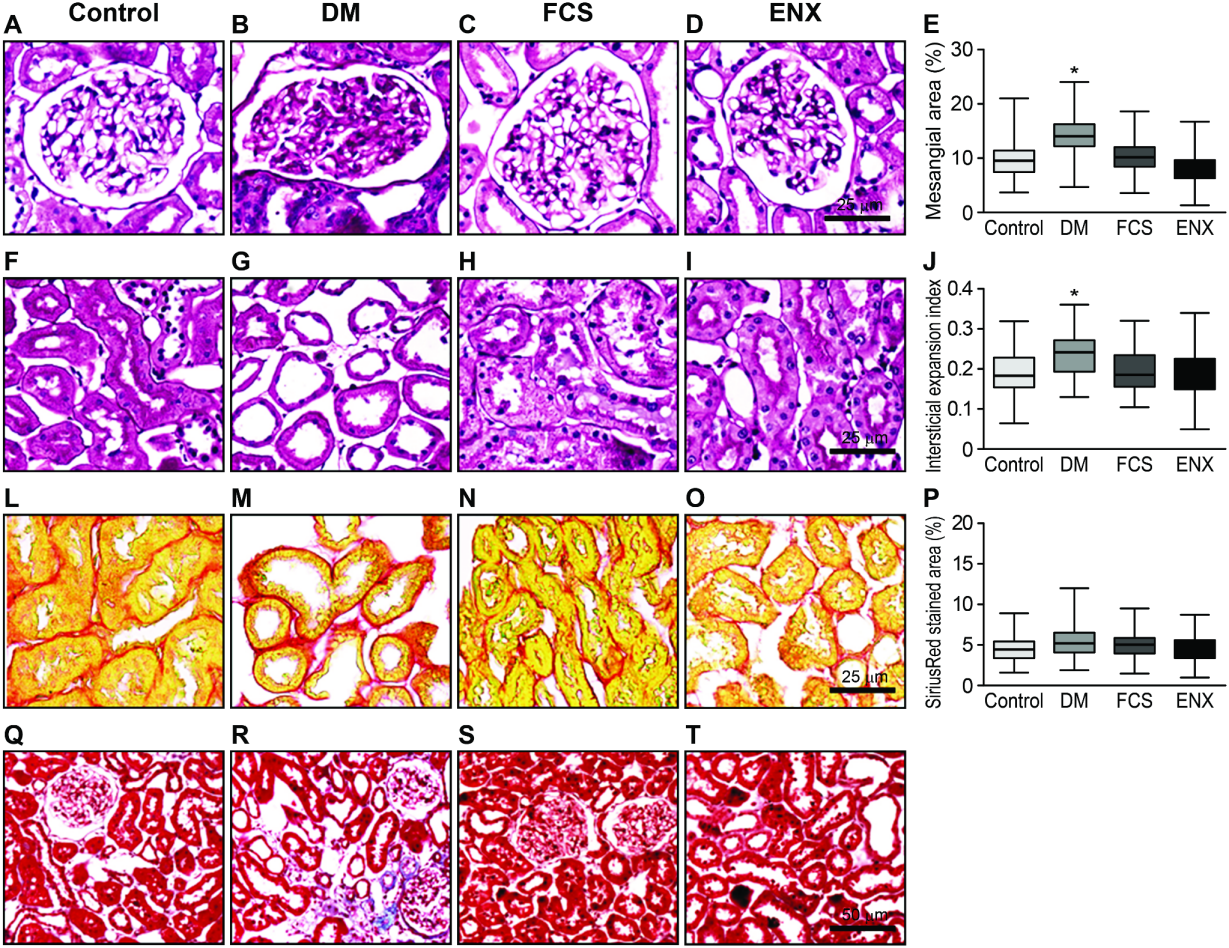
Morphological aspects of the mesangial axis and tubulointerstitial area in the experimental animals at the end of the study. A–D show PAS-stained glomerular photomicrographs with a significant increase in the mesangial area in the DM group (B) compared to the control group (A) and no increase in the DM groups treated with FCS (C) or ENX (D). In E, a semi-quantitative analysis demonstrated a 1.4-fold increase in the mesangial area in the DM group compared to the other groups. In F–I, PAS-stained renal sections show expansion of the interstitial area with tubular dilation in the DM group (G), compared to the control animals (F) and GAG-treated groups (H and I). In J, the semiquantitative analysis shows a discrete but significant expansion in the DM group. In L–N, the Sirius Red staining area shows a trend toward increased deposition of collagen fibers in the interstitial area in the DM animals (M) compared to the other groups but without statistical significance, as shown in (O). In N–P, Masson’s trichrome staining depicts the expansion of the interstitial area in the DM rats due to edema and tubular dilation compared to the treated groups. The data represent the means ± SEMs. *p<0.001, **p<0.0001. A–N, bar = 25 µm; N–P, bar = 50 µm.

At 20 weeks, an expansion of the mesangial axis was evident in the DM group compared to the control animals, as shown by a 1.4-fold increase in the PAS-positive glomerular tuft area. The FCS- and ENX-treated animals exhibited preservation of the mesangial area compared to the DM group (Table 2, Fig. 2, panels A–E, *p<*0.001). We did not find a significant prevalence of sclerotic lesions in the DM group. There was a mild but statistically significant expansion of the interstitial area in the diabetic animals compared to the control and GAG-treated animals (Table 2, Fig. 2, panels F–J, *p<*0.0001). Masson’s trichrome staining depicts the expansion of the interstitial area in the DM rats due to edema and tubular dilation compared to the treated groups (Fig. 2, panels Q–T, descriptive analysis), but without an increase in collagen deposition, as quantified by Sirius Red–stained collagen fibers in the DM group compared to the other groups (Table 2, Fig. 2, panels L–P NS).

**Table 2:** Effects of GAGs treatment on morphological and immunohistochemical parameters.

Parameter	Control	DM	FCS	ENX	*p* value
Mesangial expansion	9.5±2.8	14.9±3.01	10.27±2.6	8.19±2.5	<0.001*
Tubulointerstitial area	0.18±0.05	0.23±0.05	0.19±0.04	0.18±0.05	<0.0001*
Collagen deposition	4.8±1.4	5.3±1.7	5.1±1.5	4.9±1.4	0.068
Macrophages (x 10^−1^)	0.4±0.05	1.2±0.01	0.57±0.05	0.51±0.06	<0.0001*
TGF-β (glomerular)	0.7±0.1	4.6±0.3	1.2±0.15	1.9±0.2#	<0.0001*
TGF-β (tubulointerstitium)	0.02±0.06	0.67±0.09	0.015±0.09	0.018±0.02	<0.0001*
Nestin	7.6±0.5	2.8±0.3	6.7±0.36##	5.6±0.34**	<0.001*
Heparanase-1 positivity	1.1±0.07	2.23±0.1	1.3±0.1	0.86±0.05	<0.0001*
GBM heparan sulfate loss	0.5±0.1	2.68±0.1	2.65±0.06	2.63±0.07	<0.0001^&^

The results are presented as the means ± SEMs and expressed as the percentage of the PAS-positive area for mesangial expansion; the percentage of the area not occupied by tubules, vessels, or glomeruli for tubulointerstitial area; the percentage of the Sirius Red-positive area for collagen estimation, and the positive surface density area for macrophage, TGF-β, and nestin quantification. For heparanase-1 and heparan sulfate quantification, the results are expressed as a semi-quantitative score.

*DM vs. all of the groups;

# *p<*0.001 ENX vs. control,

**p<0.01 ENX vs. Control,

## *p<*0.05 FCS vs. ENX, & all of the diabetic groups vs. control.

### Macrophage (ED-1), TGF-β, and nestin surface density quantifications

**Figure 3:**
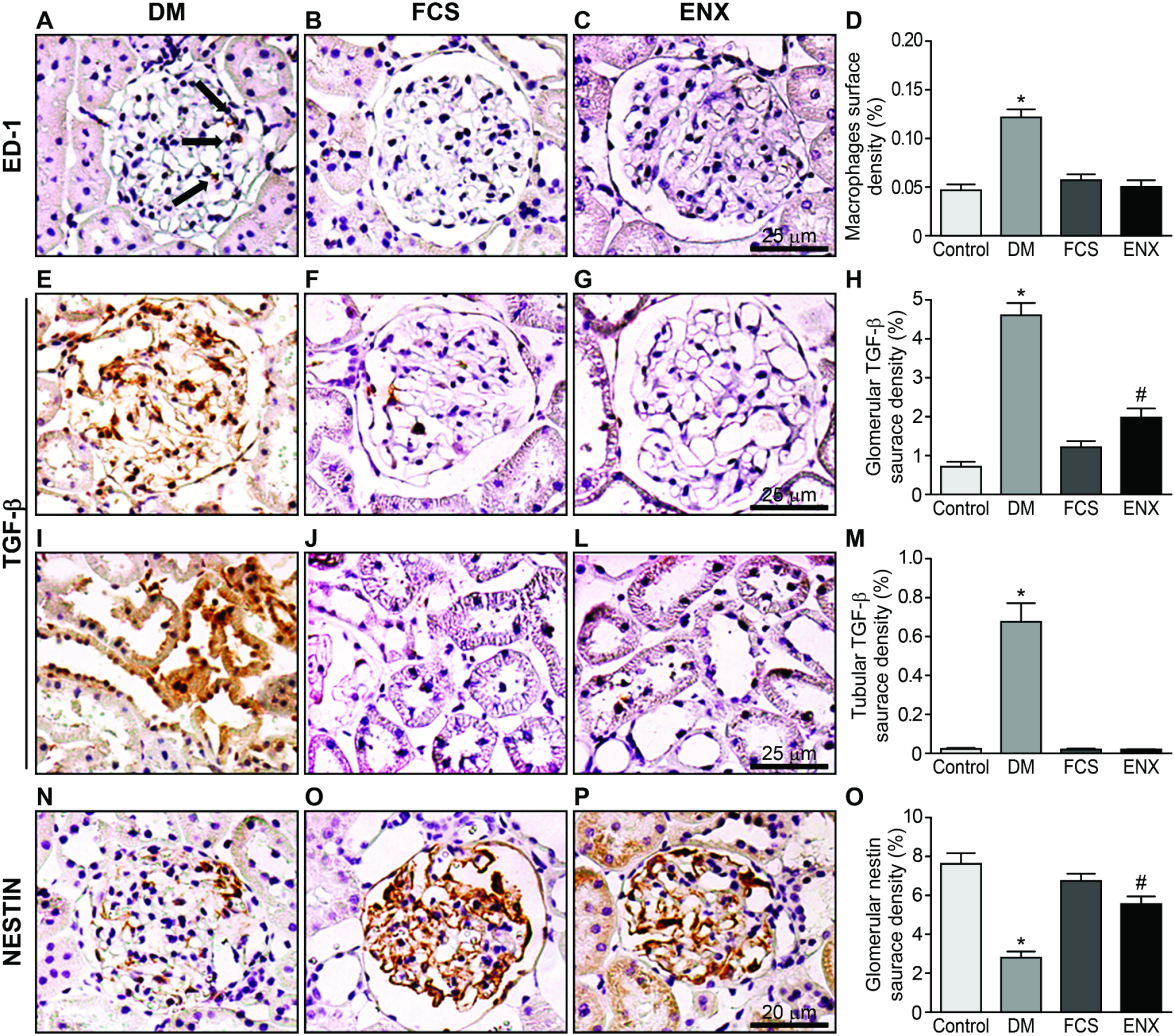
Immunohistochemical analysis at the end of the experiment. From A–C, representative photomicrographs of ED-1–stained glomerular sections showing macrophage infiltration (arrows) in the DM glomeruli (A) and indicating an increased micro-inflammatory response in the diabetic milieu compared to the normal glomeruli in the control (not shown) and FCS- (B) and enoxaparin- (C) treated animals. D shows a semi-quantitative analysis of the ED-1 surface density. E–G show photomicrographs of the TGF-β–stained glomerular sections, and I–L show the tubulointerstitial area with increased localization of TGF-β in the DM group compared to controls (not shown) and both GAG groups. H and M demonstrate a semi-quantitative analysis of the TGF-β density, confirming the histopathological findings. N–P show reduced expression of nestin in podocytes from the DM group (N) compared to a significant preservation of nestin expression in the DM groups treated with FCS (O) or ENX (P). Q shows a semi-quantitative analysis of glomerular nestin density. The data represent the means ± SEMs. *p<0.0001 DM vs. all of the groups, #*p<*0.001 ENX vs. Control, **p<0.01 ENX vs. Control, ##*p<*0.05, ENX vs. FCS. Bar = 25 µm.

Macrophage infiltration, as revealed by ED-1 expression, was significantly increased in the DM group compared to the control group (1.2±0.01×10^−1^% vs. 0.4±0.05×10^−1^%, *p<*0.0001) and to both treatment groups (0.57±0.05×10^−1^% and 0.51±0.06×10^−1^% in the FCS and ENX groups, respectively; Table 2, Fig. 3, panels A–D, *p<*0.0001). TGF-β, a pro-sclerotic growth factor with a relevant role in the pathogenesis of diabetic nephropathy, had significantly higher expression in the DM groups compared to the control group, both in the glomeruli (4.6%±0.3% vs. 0.7%±0.1%; Table 2, Fig. 3, panels E–H, *p<*0.0001) and the interstitial area (0.67%±0.09% vs. 0.02%±0.06%; Table 2, Fig. 3, panels I–M, *p<*0.0001). Again, this change was prevented by FCS or ENX treatment (1.2%±0.15% and 1.9%±0.2% of the glomerular area, respectively; *p<*0.0001; 0.015%±0.09% and 0.018%±0.02% of the interstitial area, respectively, *p<*0.0001). The expression of nestin was significantly reduced in the podocytes from DM rats compared to the control animals (2.8%±0.3% vs. 7.6%±0.5%, respectively; Table 2, Fig. 3, panels N–O, *p<*0.001), and a significant preservation of expression in the FCS and ENX groups was observed (6.7%±0.36% and 5.6%±0.34%, respectively, *p<*0.001).

### Heparanase-1 and GBM heparan sulfate quantifications

**Figure 4:**
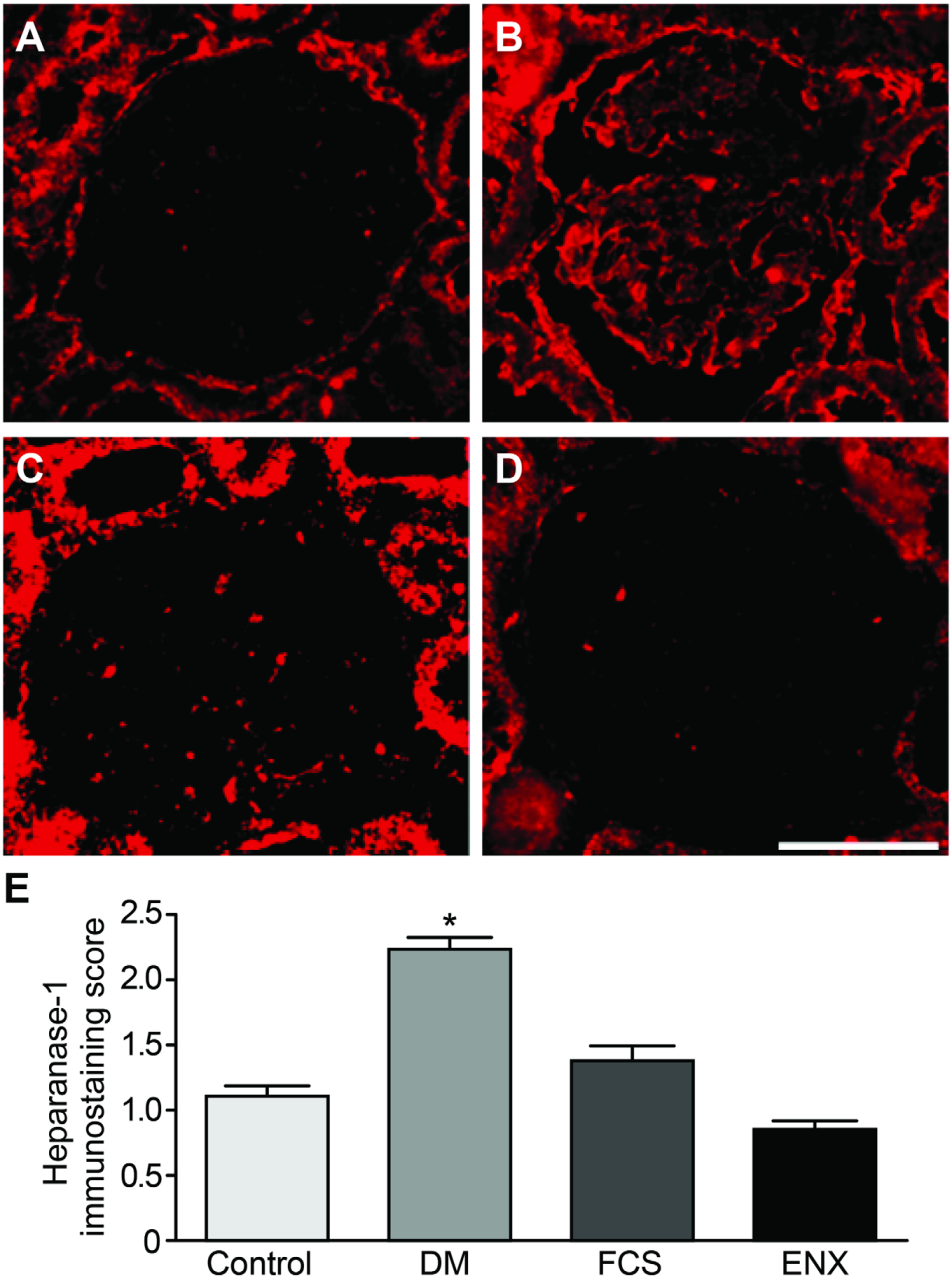
The GAGs effectively reduced heparanase-1 expression in the glomeruli of treated DM rats. From A–D, immunofluorescence photomicrographs of heparanase-1–stained renal glomerular sections with an evident increase in expression in the DM (B) animals that was prevented by FCS (C) and ENX (D) administration, as confirmed by a semiquantitative scoring system (E). The data represent the means ± SEMs. *p<0.0001 vs. groups; #*p<*0.01 vs. ENX. Bar = 25 µm.

**Figure 5:**
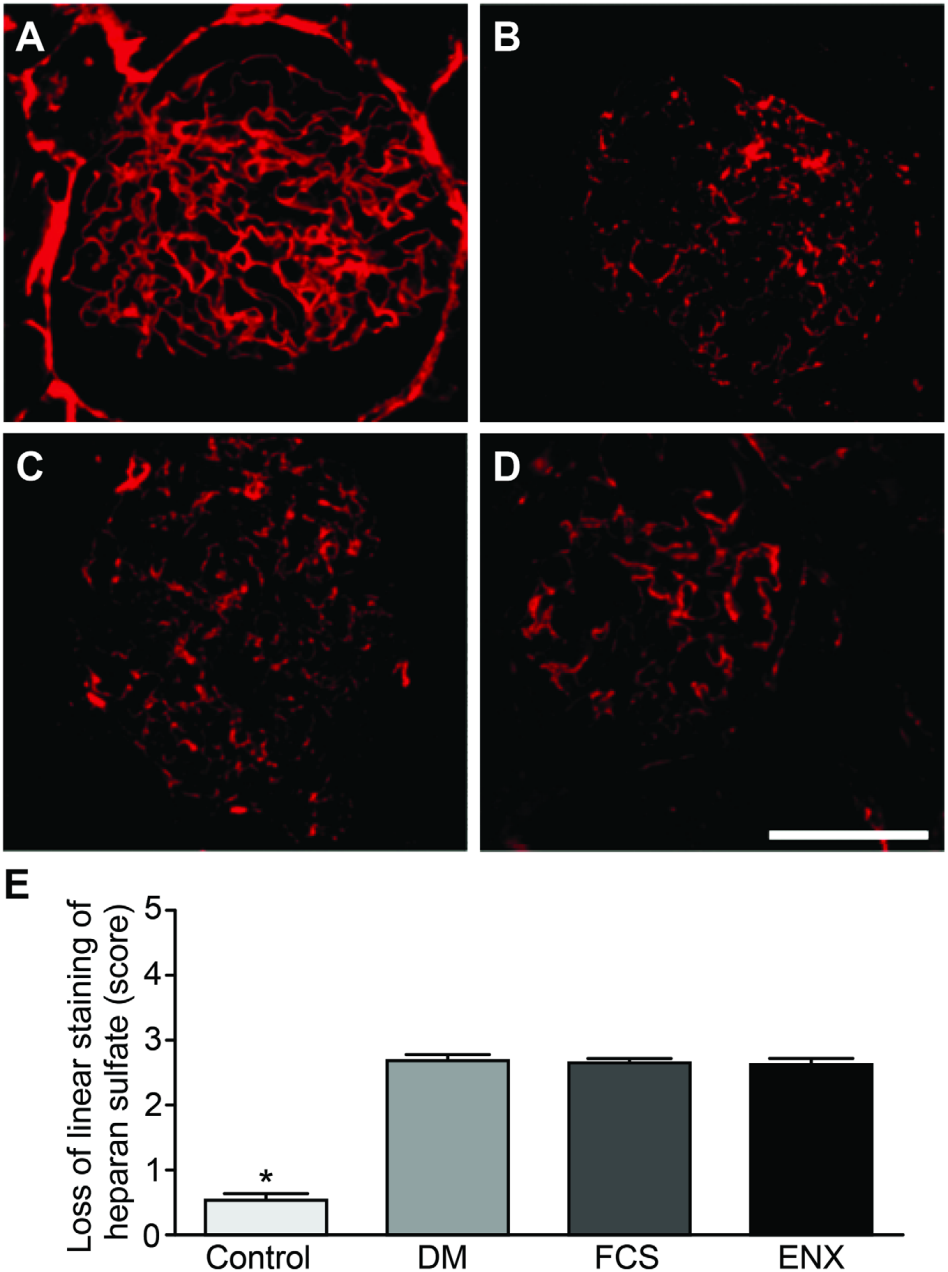
A diabetic state promoted a uniform loss of linear heparan sulfate staining in the GBM independent of the treatment. From A–D, the representative immunofluorescence photomicrographs of the heparan sulfate epitope JM-403 in the renal glomerular sections. In the control group (A), a normal linear distribution of heparan sulfate was identified in the GBM, whereas in all of the DM groups (B–D), independent of the treatment, a decreased presence of heparan sulfate in the GBM (as recognized by the JM403 epitope) was evident. This result was confirmed using a semiquantitative scoring system (E). The data represent the means ± SEMs. *p<0.0001 vs. groups. Bar = 25 µm.

To test the hypothesis that the reduced GBM HS content in DN would be prevented by treatment with GAGs and be correlated with heparanase-1 expression, the reactivity of the JM403 and HPA-1 M-45 antibodies was evaluated. Increased expression of heparanase-1 in the glomeruli from the DM group was observed compared to the control group (scores of 2.23±0.1 and 1.1±0.07; Table 2, Fig. 4, *p<*0.0001), with a consistent reduction after FCS (1.3±0.1) or ENX (0.86±0.05, *p<*0.0001) treatment. Surprisingly, we observed a uniform reduction in HS expression in the GBMs in all of the diabetic animals (loss of heparan sulfate domains as recognized by the JM403 score of 2.68±0.1 in the DM group, 2.65±0.06 in the FCS group, and 2.63±0.07 in the ENX group; Table 2, Fig. 5, NS) compared to the preservation of linearity in the control animals (score of 0.5±0.1, *p<*0.0001 vs. the other groups).

### Gene expression analysis for proteoglycan core proteins and glycosaminoglycan modifying/degrading enzymes

**Figure 6:**
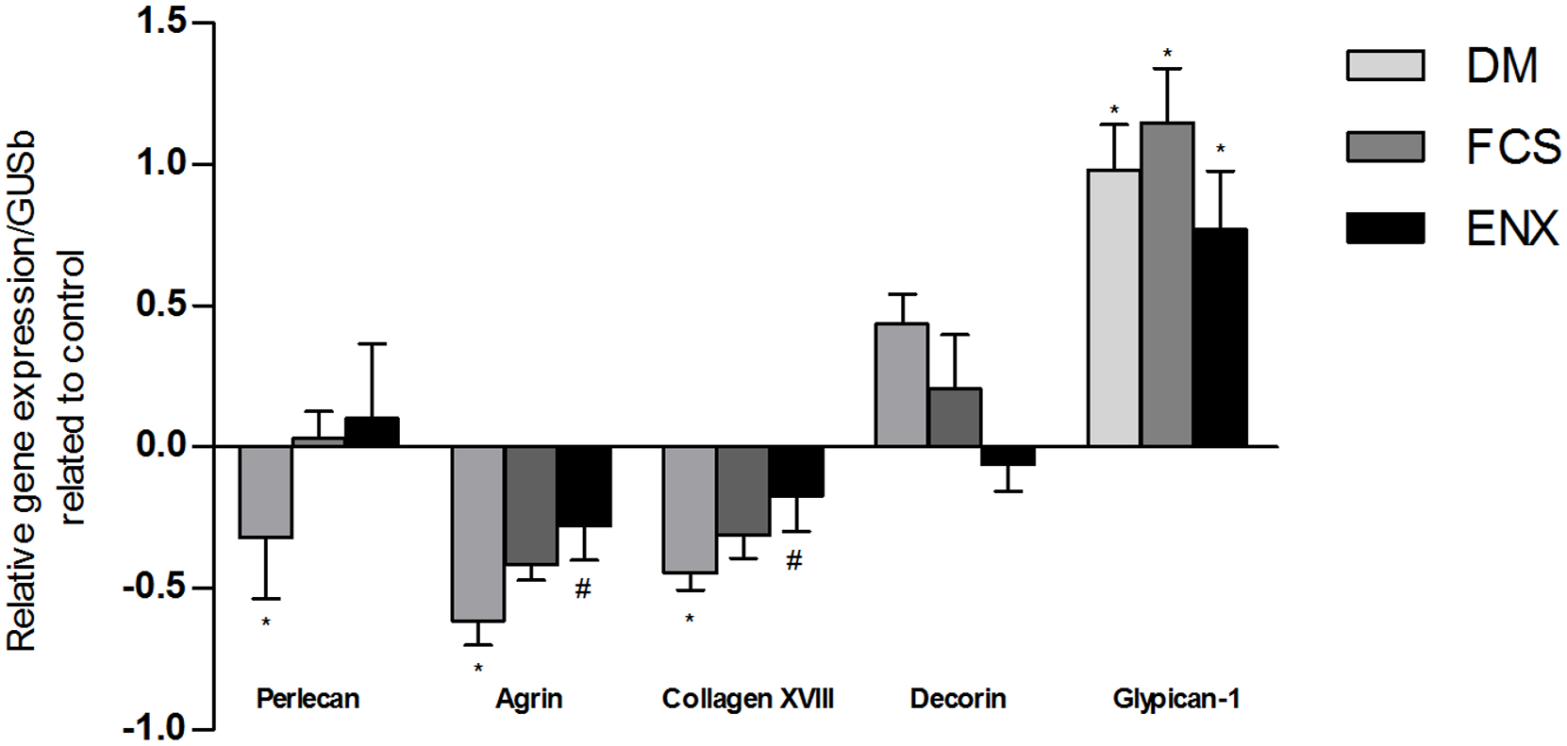
Gene expression of proteoglycan core proteins in DM and GAGs treated groups related to control. Gene expression of: agrin, the major GBM proteoglycan; perlecan and collagen XVIII, predominantly mesangial proteoglycans; decorin, a small rich-leucine proteoglycan and glypican-1, a cell suface associated proteoglycan. Gray columns, DM; dark gray, FCS and black, ENX. *p<0.01 vs. control; #*p<*0.05 vs. DM.

The expression of agrin, the major GBM PG, was downregulated by 61%±8% (*p<*0.001) in DM animals compared to controls. The perlecan and collagen XVIII core proteins mRNA levels were also downregulated in this group (−32%±2.2% and −44±5%, respectively, *p<*0.01). ENX treatment significantly attenuated agrin and collagen XVIII downregulation (*p<*0.01, Fig. 6), and a non-significant trend for the restoration of perlecan expression was also observed. FCS treatment demonstrated a trend to restore these PG expression levels, but this trend was not statistically significant (Fig. 6). We did not detect a significant difference in the gene expression of syndecan-1 or versican, while there was a non-significant trend to the increased gene expression of decorin core protein in the DM group (+43%±11%), compared to the control group, while the FCS and ENX groups revealed reduced expression (+20%±19% and −6±9%, respectively; Fig. 6, NS). There was a significant gene overexpression of glypican-1 core protein in all of the diabetic groups compared to the control (*p<*0.01, Fig. 6).

**Figure 7:**
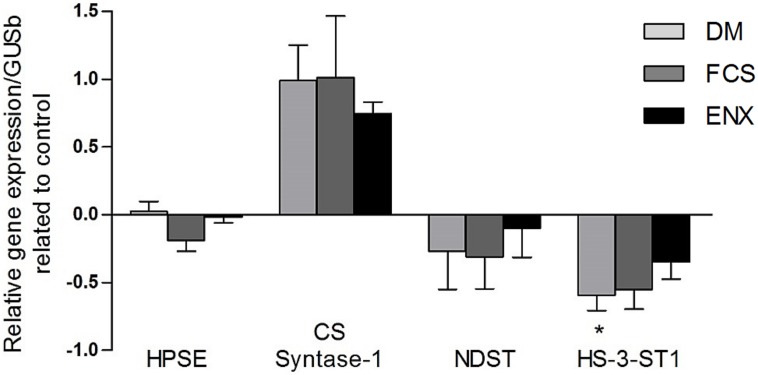
Gene expression analysis for glycosaminoglycan modifying/degrading enzymes in DM and GAGs treated groups related to control. Gray columns, DM; dark gray, FCS and black, ENX. *p<0.05 vs. control. HPSE, heparanase-1; CS, chondroitin sulfate; NDST-1, N-deacetylase/N-sulfotransferase; HS-3-ST-1, heparan sulfate-3-O-sulfotransferase 1.

Treatment with GAGs produced differing effects on expression of enzymes involved in their synthesis, chemical modification and degradation. Treatment with FCS reduced heparanase-1 gene expression by 19%±8% compared to DM group, while treatment with ENX had little effect (Fig. 7). The gene expression of two GAG enzymes important for heparan sulfate assembly and biosynthesis, N-deacetylase/N-sulfotransferase and heparan sulfate-3-O-sulfotransferase 1 were downregulated in the DM group (−27%±8.2% and −60%±9.1%, respectively, *p<*0.05, Fig. 7). These enzymes demonstrated a non-significant trend towards increased expression with ENX treatment (−10%±8,9% and −35%±8,7%, respectively), while FCS treatment had no effect. The expression of chondroitin sulfate synthase-1 was increased uniformly in all of the DM groups (Fig. 7). Due to the small sample size, some of the trends described did not reach statistical significance.

## Discussion

The therapeutic potential of GAG-based drugs has been well described in several experimental models of renal disease, particularly in DN [24]–[28]. In the present study, we tested a distinct GAG formulation, FCS, in a murine model of STZ-induced DN. Our results clearly showed the anti-inflammatory and anti-proliferative effects of GAG treatment. These effects were renoprotective, despite ongoing hyperfiltration and without affecting glycemic or blood pressure control. As we have previously shown, FCS has no anticoagulant effect at the current dosage [37], [38]. In fact, there was no hemorrhagic event during the study period.

The 1.4-fold increase in the mesangial axis in the DM animals correlated with an enhancement of TGF-β glomerular expression and the microinflammatory milieu produced by macrophage infiltration. TGF-β plays a pivotal role in DN, resulting in matrix deposition and glomerulosclerosis [2], [25]. Increased TGF-β expression in mesangial cells activated by phorbol 12-myristate 13-acetate (PMA), a TGF-β activator or cultured under hyperglycemic conditions has been described, and this effect has been shown to be inhibited by GAG-based drugs [25], [28]. Interestingly, Bacilieri et al. [29] showed that chondroitin sulfate inhibited stimulated, but not basal, TGF-β expression in mesangial cells stimulated with PMA. In contrast, heparin and derived compounds blocked TGF-β expression both at baseline and under PMA stimulation, suggesting that chondroitin sulfate would confer a more specific TGF-β blockade under pathological conditions.

In a finding similar to our results, GAG-based drugs were shown to significantly reduce macrophage infiltration and TGF-β expression in puromycin nephrosis [23]. In a mouse model of renal fibrosis [38], we have previously shown that FCS prevents TGF-β expression and macrophage infiltration, possibly through its potent P- and L-selectin blocking activity [37]. Furthermore, the enhanced heparanase-1 activity observed in DM animals could contribute to chronic low-grade inflammation through macrophage activation [41]. Diabetic mice with a null heparanase-1 gene have been shown to display significant reductions in macrophage infiltration and TGF-β expression compared to wild-type mice [18].

Because the prevention of extracellular matrix deposition does not readily explain the reduction in albuminuria in early DN, we searched for GFB alterations. Podocyte detachment is an early event in microalbuminuric diabetic patients [42] and several mechanisms in the diabetic milieu could contribute to this finding. These include hyperfiltration, angiotensin II-mediated nephrin downregulation, advanced glycation, oxidative stress, and TGF-β-induced podocyte apoptosis [43]. Some of these stimuli, such as angiotensin II, oxidative stress and aldosterone, stimulate heparanase-1 production by podocytes [44], and podocyte apoptosis induced by oxidative stress had been prevented by treatment with heparin, a heparanase-1 antagonist [45]. Furthermore, a hyperglycemic medium has been shown to enhance heparanase-1 expression in cultured podocytes, decreasing GBM HS content, while heparin and a heparanase-1 antagonist reversed these findings [15]. Therefore, TGF-β and heparanase-1 inhibition using GAG treatment should have protective effects against podocyte damage in DN.

The role for the negative charge of the GBM in glomerular permselectivity is still debated [7], [9], [20]. Our comprehensive analysis of the gene expression of proteoglycan core proteins and enzymes involved in GAG assembly/degradation and the glomerular expression of HS and heparanase-1, revealed interesting results. The gene expression of agrin, the major GBM proteoglycan, together with the gene expression of perlecan and collagen XVIII (predominantly mesangial proteoglycans), was significantly reduced in DM animals. Conflicting evidence regarding the expression of agrin in diabetic nephropathy has been produced, with studies showing both a reduction [46] and preservation of expression [14]. However, the expression of perlecan is often reduced in DN, despite the increased mesangial matrix [26]. Treatment with GAGs significantly reversed or showed a trend towards reversal in the gene expression of these proteoglycans.

An intriguing finding was the uniform reduction of the GBM HS content in all of the diabetic animals, regardless of the GAG treatment. Recently, a body of evidence arguing against a role for GBM HS in the pathogenesis of albuminuria has emerged. Agrin- and perlecan-deficient mice, with significantly reduced GBM HS contents, have failed to show renal phenotype alterations or albuminuria [11]. Using Cre-lox technology, Chen et al. [10] created EXT-1 deficient mice. This gene encodes for exostosin glycosiltranferase-1, a co-polymerase that participates in HS assembly, which when deleted did not induce significant albuminuria. Moreover, in diabetic heparanase-1 null mice, Gil et al. [18] showed only a modest alteration in GBM HS, although there was a significant reduction of albuminuria.

Accordingly, our findings showed that GBM HS loss did not affect albumin filtration presumably because both GAG preparations prevented albuminuria and other early pathological alterations without affecting the HS content, albeit with gene expression preservation of PG core proteins. In addition, there was a discrepancy between gene expression and post-translational expression of heparanase in the DM group. We speculate that our intense regimen of insulin might have influenced the expression of heparanase. This has been previously shown in endothelial cells cultured in a high glucose medium, where the overexpression of heparanase was suppressed by adding insulin to the medium [47]. It has also been shown that insulin regulates the Sp1 nuclear transcription factor [47], and since heparanase genes possess Sp1 binding sites within their promoter regions [48], decreased mRNA expression of heparanase may be due to the regulation of Sp1 by insulin. On the other hand, the inflammatory milieu produced by macrophage infiltration might have contributed to the increased heparanase activity, as macrophages might express and produce heparanase under chronic inflammatory conditions [41]. Therefore, we speculate that a post-translational reduction in the activity of heparanase-1 under diabetic conditions with GAG treatment could have a protective role, involving mechanisms other than the preservation of the GBM HS. These include the already mentioned modulation of macrophage activity [41] and podocyte protection [45]. These seemingly contradictory findings require further elucidation. In addition, we cannot exclude the possibility of a “dilution effect” in the HS JM403 staining, due to thickening of the GBM secondary to hyperfiltration and persistent hyperglycemia, resulting in an apparent but not absolute decrease in the content of HS in all diabetic animals, despite the treatment [49].

A further interesting finding was the expression modulation of HS-3-O-sulfotransferase-1. This enzyme is responsible for an infrequent, yet important and late, modification of the HS chain (irrespective of chain size), through the addition of the 3-O-sulfate group to glucosamine residues, preferentially generating a pentasaccharide motif that binds and activates antithrombin (AT). This modified HS chain is referred to as anticoagulant HS. Glomerular epithelial cells regularly synthesize anticoagulant HS, which is distributed throughout the GBM [50]. Such HS could play important role in regulating local balance between thrombin/fibrin formation and degradation, via antithrombin activation, aside of being able to bind potential harmful effectors such as fibroblast growth factor [50]. Decreased HS-3-O-ST activity has been demonstrated in the GBM of DM patients [51], and in experimental puromycin nephrosis [52]. In this study, we detected a reduction in gene expression of HS-3-O-ST in DM animals, with GAG treatment conferring a partial restoration.

Although not statistically significant, there was an up-regulation of chondroitin sulfate synthase-1 in all of the DM groups. We speculate that it was a transcriptional response to a post-translational reduction in renal content of chondroitin/dermatan sulfate or in the degree of sulfation of chondroitin sulfate chains, which has been already described in experimental DN [53], [54]. Gene expression analysis of other important PG core proteins also revealed interesting results. There was a significant increase in the gene expression of cell surface PG glypican-1 in all of the DM groups, irrespective of treatment. Up-regulation of glypican-1 expression has previously been demonstrated in the cardiomyocytes of STZ-induced diabetic rats [55]. The authors speculated on its potential role in modulating the interaction of growth factors such as fibroblast growth factor, which is augmented in DM and is possibly implicated in the mechanism of tubulointerstitial injury in long term DM [27]. To our knowledge, this is the first study to report an increased expression of glypican-1 in renal tissues under diabetic conditions, and the relevance of this finding requires further investigation. Decorin, a small leucine-rich proteoglycan, has an important role in counteracting the profibrotic effects of TGF-β, and its levels are elevated in DN [56]. Our results show a trend toward an increased expression of decorin in DM animals, paralleled with an increased expression of TGF-β, with downregulation in both treated groups, reflecting the ability of GAG treatment to suppress TGF-β expression.

Finally, the endothelial glycocalyx in GFB permselectivity, due to its high GAG and sialoglycoprotein anionic content [3], [57], shows perturbations in diabetic mice [58] and in human DN [42]. This role makes it an attractive site for exogenous GAG interactions. Heparanase-1 has been shown to reduce the HS content in the glycocalyxes of cultured human glomerular endothelial cells and enhance transendothelial albumin traffic [59]. Apart from a significant role in GFB permselectivity, modulation of specific HS domains in the glomerular endothelial glycocalyx might play an important role in inflammatory conditions. This has been shown by Rops et al. [57], where an endothelial cell-specific NDST-1 deficiency in mice significantly ameliorated anti-GBM nephritis by reducing glomerular leukocyte influx. The protective effect of GAGs on albuminuria in DN could possibly be related to endothelial protection. The absence of such an analysis is one of the limitations of our study, and this hypothesis requires further investigation. Another limitation of our study was the limited number of animals in each group, particularly with regards to PCR analysis. This limitation may partly explain why differences between groups often did not reach statistical significance.

In conclusion, FCS and ENX attenuated the onset of albuminuria and prevented mesangial and tubulointerstitial expansion in a murine model of STZ-induced diabetic nephropathy. Furthermore, they decreased macrophage infiltration and the expression of heparanase-1 and TGF-β. There was a preservation of podocyte structure and the gene expression of the major glomerular proteoglycan core proteins, but without changing the heparan sulfate content. Important enzymes involved in GAG assembly/degradation were positively modulated by GAG treatment. FCS, the novel GAG formulation used in this study, did not show any anticoagulant effects at the dosage used. Additional research is needed to confirm whether treatment with GAGs could be a promising add-on therapy for this devastating complication of long-term DM.
